# Travel-Associated Zika Cases and Threat of Local Transmission during Global Outbreak, California, USA

**DOI:** 10.3201/eid2409.180203

**Published:** 2018-09

**Authors:** Charsey Cole Porse, Sharon Messenger, Duc J. Vugia, Wendy Jilek, Maria Salas, James Watt, Vicki Kramer

**Affiliations:** California Department of Public Health, Sacramento, California, USA (C.C. Porse, W. Jilek, V. Kramer);; California Department of Public Health, Richmond, California, USA (S. Messenger, D.J. Vugia, M. Salas, J. Watt)

**Keywords:** Zika, Zika virus, viruses, arboviruses, travel-associated illness, local transmission, global outbreak, Aedes aegypti mosquitoes, Aedes albopictus mosquitoes, vector-borne infections, zoonoses, California, United States, Mexico

## Abstract

Zika and associated microcephaly among newborns were reported in Brazil during 2015. Zika has since spread across the Americas, and travel-associated cases were reported throughout the United States. We reviewed travel-associated Zika cases in California to assess the potential threat of local Zika virus transmission, given the regional spread of *Aedes aegypti* and *Ae. albopictus* mosquitoes. During November 2015–September 2017, a total of 588 travel-associated Zika cases were reported in California, including 139 infections in pregnant women, 10 congenital infections, and 8 sexually transmitted infections. Most case-patients reported travel to Mexico and Central America, and many returned during a period when they could have been viremic. By September 2017, *Ae. aegypti* mosquitoes had spread to 124 locations in California, and *Ae. albopictus* mosquitoes had spread to 53 locations. Continued human and mosquito surveillance and public health education are valuable tools in preventing and detecting Zika virus infections and local transmission in California.

The first human cases of Zika virus infection reported from the Americas were in May 2015 from Brazil (*1*). In the span of less than a year, Zika virus spread across South America, Central America, the Caribbean, and parts of Mexico. As observed with other mosquitoborne diseases, such as dengue and chikungunya, which have spread through Central and South America and the Caribbean, travel-associated cases of Zika were reported throughout the United States, and local transmission of Zika virus was eventually detected in Florida and Texas (*2**,**3*). Because California has established and expanding infestations of *Aedes aegypti* and *Ae. albopictus* mosquitoes, the main vectors of Zika virus, and is near Mexico, to which Zika virus is endemic, the risk for autochthonous transmission of Zika virus is a concern (*4*). During 2011–2015, *Ae. aegypti* and *Ae. albopictus* mosquitoes were detected in 85 cities and census-designated places in 12 counties of California (*5*).

In California, patient testing and evaluation focused on assessment of infection in pregnant women and symptomatic patients, and assessment of potential viremia in these patients in relation to proximity to known *Aedes* mosquito infestations. To describe travel-associated Zika cases and better assess the potential threat of local Zika transmission in California, we reviewed all Zika cases reported to the California Department of Public Health (CDPH) during November 2015–September 2017. We also summarized surveillance for *Ae. aegypti* and *Ae. albopictus* mosquitoes in California and laboratory testing for Zika virus during this time.

## Methods

Zika cases were reported to CDPH by the 61 local health departments in California, either through the electronic California Reportable Disease Information Exchange (https://www.cdph.ca.gov/Programs/CID/DCDC/Pages/CalREDIE.aspx) or through paper case report forms. Cases reviewed by CDPH during November 2015–September 2017 were analyzed for type of Zika disease or infection, as defined by the 2016 Council of State and Territorial Epidemiologists (CSTE)/Centers for Disease Control and Prevention (CDC; Atlanta, GA, USA) criteria and classified as confirmed or probable (*6*). Variables examined included sex, age, race/ethnicity, country where exposure likely took place, duration of travel, symptoms, symptom onset date, and pregnancy status and outcomes.

We analyzed data by using SAS for Windows version 9.4 (SAS Institute Inc., Cary, NC, USA). For Zika case-patients with a travel duration of <6 months, we compared duration of time in Zika-affected areas between pregnant and all other case-patients by using the Kruskal-Wallis test for 2 groups (unequal variances) to retrospectively assess time at risk between these 2 groups.

California has a network of local vector control agencies that monitors distribution and abundance of *Aedes* spp. and other mosquito populations. Mosquito surveillance typically includes trapping and identifying mosquitoes. Surveillance might be augmented by submitting mosquito specimens, especially specimens collected in and around residences or workplaces of case-patients, to the Davis Arbovirus Research and Training Laboratory at the University of California (Davis, CA, USA) for Zika virus (*7*), dengue virus (DENV) (*8*), and chikungunya virus (Davis Arbovirus Research and Training Laboratory at the University of California, unpub. data) testing by multiplex quantitative reverse transcription PCR (RT-PCR), as described by CDC (*9*). Mosquitoes submitted during West Nile virus (WNV) season (June 1–October 15) are also tested for WNV, St. Louis encephalitis virus, and western equine encephalitis virus (*10*). Agencies enter mosquito surveillance data into the California Vectorborne Disease Surveillance Gateway Database (https://gateway.calsurv.org/), which is used to generate statewide data for mapping of *Aedes* mosquito locations. We used a geographic information system (ArcGIS Desktop version 10.5; Esri, Redlands, CA, USA) to generate maps that enabled spatial and temporal mapping of *Aedes* mosquito populations in relation to presumed places of residence of presumed viremic Zika case-patients. We generated latitude and longitude data by using the Gateway Database for mosquitoes and determined case-patient place of residence by using the California Reportable Disease Information Exchange.

Testing of humans for Zika virus was performed by the CDPH Viral and Rickettsial Disease Laboratory (VRDL), CDC, local public health laboratories, commercial laboratories, and blood banks. Testing for Zika virus infection was completed for appropriate tissue, serum, or urine specimens by using Zika virus nucleic acid or serologic tests. We analyzed symptomology and pregnancy status of those tested, volume of testing at the CDPH VRDL, types of tests conducted, and time from symptom onset to specimen collection date. For purposes of local transmission risk assessment, a potentially viremic patient was defined as a Zika-positive case-patient with symptom onset <7 days before or any time after return from travel to their place of residence.

## Results

### Descriptive Statistics

During November 2015–September 2017, a total of 588 travel-associated Zika cases were reported in California, including 139 infections in pregnant women, 10 congenital infections, and 8 sexually transmitted infections. Sixty-two case-patients were <18 years of age. On the basis of the 2016 CSTE surveillance case definition for Zika, 410 cases met the confirmed criteria and 178 were probable. Of these, 466 case-patients had noncongenital Zika disease with symptoms meeting the 2016 CSTE case definition for noncongenital Zika (>1 of the following: fever, rash, arthralgia, or conjunctivitis); 112 had a symptomatic noncongenital Zika infections, 6 had congenital Zika disease with Zika-associated birth defects (birth defects reported include those detected in infants infected with Zika virus before, during, or shortly after birth, including microcephaly, calcium deposits in the brain indicating possible brain damage, excess fluid in the brain cavities and surrounding the brain, absent or poorly formed brain structures, abnormal eye development, or other problems resulting from damage to the brain that affects nerves, muscles, and bones, such as clubfoot or inflexible joints, and confirmed hearing loss); and 4 had congenital Zika infections with no Zika-associated birth defects (*6*).

A total of 66% (391/588) of case-patients were female; median age of case-patients was 35 years (range <1–89 years). Of persons with reported ethnicity, 69% (306/443) were Latino/Latina. For the 139 women pregnant at the time of diagnosis, median age was 27 years (range 14–44 years), and 78% (87/111) of those with reported ethnicity were Latina.

**Figure 1:**
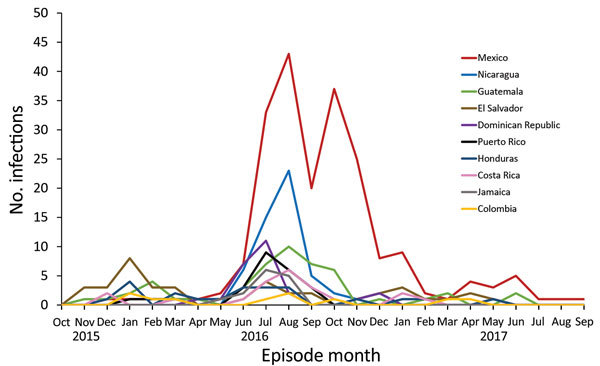
Number of human Zika virus infections in residents, by month and year of onset and country of travel (top 10 countries shown), California, USA, October 1, 2015–September 1, 2017. Month was determined by date of symptom onset for symptomatic persons or specimen collection date for asymptomatic persons.

Of 570 case-patients who contracted Zika virus while traveling outside California, most case-patients reported travel to Mexico (36.4%), Central America (34.3%), or the Caribbean (13.1%). The top 10 countries and territories for travel were Mexico (36.4%), Nicaragua (9.6%), Guatemala (8.4%), El Salvador (7.0%), Dominican Republic (4.4%), Puerto Rico (4.4%), Honduras (3.9%), Costa Rica (3.7%), Jamaica (2.5%), and Colombia (1.8%). The timeline for travel-associated Zika cases reported in California mirrored the spread of the outbreak across the Americas (Figure 1); the number of case-patients with travel to Mexico increased substantially starting in June 2016 as the number of Zika cases reported in Mexico steadily increased.

Of 570 case-patients who traveled, 79 (13.9%) lived in their country of exposure for >6 months before coming to California, where they were subsequently tested for Zika virus. When we excluded these 79 persons, women who were pregnant at the time of Zika diagnosis had a significantly (p = 0.03) longer travel duration (median 14 days [range 1–153 days]) than all other Zika case-patients (median 11 days [range 1–137 days]).

**Figure 2:**
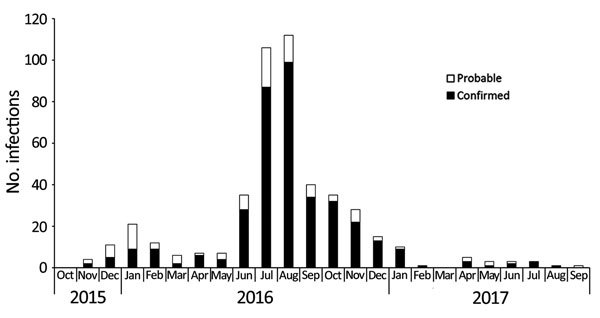
Confirmed and probable symptomatic Zika virus infections, by symptom onset month and year, California, USA, October 2015–September 2017.

For 466 case-patients with symptoms, rash was the most common (89.0%, 415), followed by arthralgia (62.5%, 291), fever (60.1%, 280), myalgia (36.9%, 172), and conjunctivitis (35.0%, 161). A rash without any other symptom was seen in 49 (10.5%) case-patients. For those case-patients with >1 symptom, the most common combination of symptoms, reported by 13% of case-patients, was rash, arthralgia, and fever. Seven case-patients were hospitalized for a median of 3 (range 1–8) days. On the basis of symptom onset date, the number of Zika cases reported in California in 2016 increased from June through August and then decreased through November (Figure 2).

Of 139 women who were pregnant at the time of Zika diagnosis, 120 had completed their pregnancies by September 1, 2017: 114 with live births and 6 with fetal losses. Fourteen women were still pregnant, and the status of 5 women was unknown. For live births, 90 (78.9%) infants were tested for Zika virus at or shortly after birth; 84 (73.7% of live births) infants showed negative results for Zika virus by nucleic acid and IgM tests, and 6 (5.3% of live births) showed positive results. Of the remaining 24 live births, 7 infants were not tested, and the testing status of 17 infants was unknown. In addition to the 6 congenitally infected infants that were born to Zika virus–positive mothers, 4 additional infants whose mothers were exposed to Zika virus but showed negative results by nucleic acid or IgM tests were positive for Zika virus.

Eight infants were born in California with Zika-associated birth defects. Of these infants, 2 were negative and 6 were positive for Zika virus by PCR and IgM test. Both Zika virus–negative infants had mothers who were positive for Zika virus, and 3 of the Zika virus–positive infants had mothers who had negative results for Zika virus.

### Mosquito and Human Case Surveillance

During January 1, 2016–September 1, 2017, we detected 78 new locations for *Ae. aegypti* mosquitoes and 25 new locations for *Ae. albopictus* mosquitoes, for a total of 133 cities or census-designated places for *Ae. aegypti* mosquitoes and 56 for *Ae. albopictus* mosquitoes, an increase of 142% for *Ae. aegypti* mosquitoes and 81% for *Ae. albopictus* mosquitoes in 20 months. In 2017, *Ae. aegypti* mosquitoes were detected in 12 counties and *Ae. albopictus* mosquitoes in 5 counties, including 2 new counties containing *Ae. aegypti* mosquitoes in the Central Valley (*11*).

**Figure 3:**
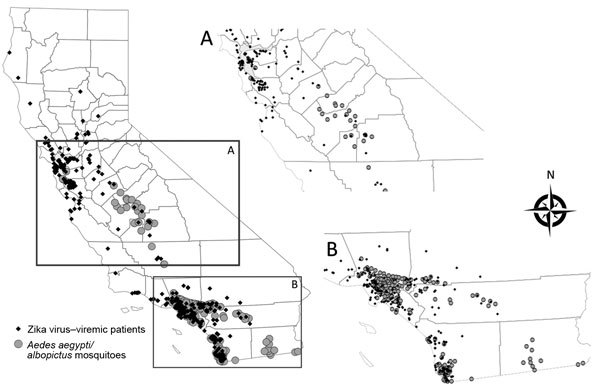
Locations where *Aedes* spp. mosquitoes were detected and residences of possibly viremic case-patients infected with Zika virus, central (A) and southern (B) California, USA, October 2015–September 2017. Insets show larger views of corresponding region.

As of September 1, 2017, a total of 13,499 *Ae. aegypti* mosquitoes and 2,719 *Ae. albopictus* mosquitoes had been tested by Davis Arbovirus Research and Training for Zika virus, chikungunya virus, and DENV. None of these mosquitoes were positive for these arboviruses, although 5 pools of *Ae. aegypti* mosquitoes and 1 pool of *Ae. albopictus* mosquitoes were positive for WNV. Of the 588 case-patients reported who had Zika virus infections, 435 (74.6%) were likely viremic while in California. Of those viremic case-patients, 279 (64.1%) were also residents of California counties where *Ae. aegypti* or other *Aedes* spp. mosquitoes have been detected; their co-location was more common in southern California (Figure 3).

### Laboratory Testing

Although the VRDL performed most (58.7%; 345/588) testing for Zika-positive cases in California, commercial laboratories accounted for 17.5% (103/588), local health departments for 13.1% (77/588), and CDC for 9.5% (56/588). Seven Zika cases reported in California were identified through blood bank screening. Most testing at VRDL was performed for asymptomatic pregnant women (7,795 asymptomatic pregnant women/11,603 total patients; 67.2%). Eighty (1.0%) of these asymptomatic pregnant women were positive for Zika virus by quantitative RT-PCR (1 woman) or IgM test and plaque-reduction neutralization test (PRNT) (79 women).

Of the 120 completed pregnancies for women who were infected with Zika virus while pregnant, 45 placental tissues (including placenta, membrane, and umbilical cord) were sent to CDC for testing. Zika virus was detected by RT-PCR in placental tissues of 8 women. Detection of Zika virus in the placental tissue provided confirmatory testing for 5 of these women (3 were already confirmed by serum PRNT).

Of the 410 confirmed Zika cases reported in California, 319 (77.8%) case-patients had Zika virus detected by nucleic acid tests in serum, urine, or placental tissue, and the other 91 were confirmed by detection of neutralizing antibodies to Zika virus (and not DENV). For symptomatic case-patients, the median time from illness onset to specimen collection was 5 days (range 1–194 days). For cases confirmed by serum or urine nucleic acid tests, the time to collection was shorter, with a median of 3 days (range 1–33 days), than for PRNT, with a median of 16 days (range 1–194 days).

## Discussion

Since the global Zika outbreak began in South America in 2015, many travel-associated Zika cases have been documented in California, including infections in pregnant women, congenital infections, and sexually transmitted infections. With the establishment and continuing spread of *Ae. aegypti* and *Ae. albopictus* mosquitoes in California, prevention of local transmission of Zika virus has been and continues to be a public health priority. In working to identify possible local transmission, CDPH used the data for travel-associated Zika cases described in this article to develop our Zika testing prioritization. Although CDC recommended specific criteria for travel-associated Zika virus testing, different criteria were needed when testing persons without travel history, especially when the number of confirmed Zika cases was increasing in California and local Zika virus transmission was reported in Florida (*2*). The goal of such testing was to identify anyone who potentially had Zika virus, without testing large numbers of persons at low risk.

CDPH subsequently provided criteria for local health departments in California to consider in evaluating whether suspected persons without travel history should be considered for Zika virus testing, including factors that could increase risk for local transmission, as well as signs and symptoms most suggestive of Zika. For example, CDPH allowed that, for counties where *Aedes* mosquitoes have been detected, Zika virus testing could be offered to persons who live in an area containing *Aedes* spp. mosquitoes and who came to their healthcare provider with a maculopapular rash and 1 other symptom consistent with Zika (fever, arthralgia, or conjunctivitis), without an alternative explanation, such as a drug reaction or other infection. Rash was recommended as the primary criterion in this setting because nearly 90% of Zika case-patients had a rash. This allowance for Zika virus testing for persons with no travel or sexual exposure was used in some counties in California and identified several persons suspected of having Zika who were tested, all of whom showed negative results. This testing allowance would not be appropriate in areas that did not contain *Aedes* spp. mosquitoes and is being reconsidered as the number of Zika cases has decreased.

Although California health officials did not identify any episodes of local Zika virus transmission, our data indicate that large numbers of likely viremic travelers returned to areas containing *Ae. aegypti* and *Ae. albopictus* mosquitoes, especially in southern California, as has also been found for dengue and chikungunya (*4*). This overlap of viremic travelers and *Aedes* spp. mosquito vectors potentially increases the risk for local transmission and will continue to be a public health concern requiring ongoing mosquito and human case surveillance. CDPH works closely with local health departments and vector control agencies to prepare for the potential of a locally transmitted outbreak. The close coordination of mosquito control programs in California with programs of local health departments, the common use of air conditioning or window screens by residents, and the variable distribution of *Aedes* spp. mosquitoes in some affected counties in California would likely limit the extent of a local outbreak should it occur. Nonetheless, knowledge of co-located vector mosquitoes and infected returned travelers is needed to aid in rapid investigation of any suspected locally transmitted case(s) and to limit potential spread.

Mosquito seasonality influences risk for local transmission, and although *Aedes* spp. mosquitoes can be found year-round in California, they are most abundant from June through November, typically peaking in September and October (*5*). Large numbers of potentially viremic case-patients returned to California during June–November 2016 (Figure 2), corresponding with the season of high *Aedes* spp. mosquito activity in California. This seasonality also reflects *Ae. aegypti* mosquito activity in northern Mexico, where *Ae. aegypti* mosquitoes are abundant from August through November.

Similar to the chikungunya outbreak in the Americas that began in 2013 and rapidly peaked in most locations before decreasing (*12*), the number of Zika cases is now decreasing. This decrease in Zika cases has been observed both in countries reporting local transmission and in the number of infected returned travelers reported in the United States and in California (*13*). Although the level of Zika virus transmission has decreased, many countries, including Mexico, have continued to report moderate levels of local Zika virus transmission (*14*). Given the large number of travelers between Mexico and California, it is critical that Zika prevention messaging, surveillance, and outreach continue, especially as it pertains to women traveling while pregnant.

The large volume of testing for asymptomatic pregnant women reinforces that potential Zika virus exposure incidents were occurring in high numbers even with extensive provider education and public health messaging in California and nationally. Women who were pregnant at the time of their Zika diagnosis had a longer duration of travel in their exposure country than all other case-patients. Because most infected pregnant women were Latina, it is possible that many of them had traveled to visit family and therefore had longer stays. Given the health risk to pregnant women and their fetuses, this finding is of great concern. We need to ensure that English- and Spanish-language public health messaging about risks of travel or travel of sexual partners to Zika-affected countries continues to reach pregnant women and their healthcare providers. Although a decrease in reported travel-associated Zika cases was observed in California in March 2017, we did not detect a decrease in specimens submitted for asymptomatic pregnant women to VRDL until August 2017.

Laboratory testing for Zika virus has proved challenging throughout the outbreak. Results of assays were difficult to interpret because serologic cross-reactivity with other flaviviruses, especially DENV, was common (*15*). Detection of neutralizing antibodies against Zika virus and DENV was observed for 178 probable Zika case-patients reported in California. Thus, the specific flavivirus of the infection in these case-patients could not be determined. In addition, Zika IgM has been reported to persist in serum, making timing of infection difficult to determine (*16*). The discordant testing results observed in the mother/infant pairs were equally challenging, suggesting that a negative test result could rarely rule out a Zika virus infection. All these factors, in addition to the difficulty of determining the date of exposure for many case-patients, especially for women who lived in the area of exposure for an extended time, made the interpretation of negative results problematic and created challenges for ensuring that affected infants received appropriate follow-up care.

Our study and data interpretation have several limitations. First, the data included only case-patients who were positive for Zika virus, not case-patients who were negative but had been potentially exposed to Zika virus. Analysis of such persons who were negative for this virus but had potentially been exposed would be helpful to further delineate risk and discriminate symptoms. However, negative results, particularly from commercial laboratories, often have limited associated clinical and demographic data. Second, some dengue cases might be misclassified as Zika cases because of cross-reactivity and nonspecific binding in available serologic assays. Given the large percentage of case-patients in California with previous exposure to flaviviruses, especially DENV, there is potential for false-positive interpretation of PRNT results. All case-patients with neutralizing antibodies against DENV and Zika virus were classified on the basis of the CSTE case definition as having Zika because of the higher risk during pregnancy from exposure to Zika virus. In addition, low pretest probability, especially in asymptomatic persons, increases the risk for misclassification because of type I errors (false-positive results). Third, an estimated 80% of persons infected with Zika virus are asymptomatic (*17*), making it difficult to determine when, where, and how many potentially viremic persons are returning to California. Fourth, there is a clear testing bias toward pregnant and reproductive-age women, which skews demographic data.

Although Zika virus transmission and Zika case numbers have decreased across the Americas, we expect to see continued, limited, local transmission in some affected countries. Thus, there is still a risk for pregnant women and all those who travel to these countries, and it is necessary that prevention messaging remains targeted and operative. Healthcare providers should continue to be suspicious of returning travelers with rash, fever, conjunctivitis, or arthralgia, particularly when other diagnoses have been ruled out. The expansion of *Ae. aegypti* and *Ae. albopictus* mosquitoes into 12 counties in California, especially along the southern border region, increases the risk for local Zika transmission in California. The large percentage of potentially viremic travelers returning to areas that contain *Aedes* spp. mosquitoes, in addition to an unknown number of returned travelers who are asymptomatically infected but not detected, makes the risk for local transmission a continuing threat, albeit low, in California. Zika has complicated disease manifestations and transmission dynamics, such as sexual and congenital transmission, which are not typically observed for other arboviruses. It is vital that we apply the public health lessons learned during the Zika outbreak to prepare for complexities that might arise during future epidemics of emerging and reemerging arboviruses.
